# Mendelian randomization reveals plasminogen as a common therapeutic target for myocardial infarction and atrial fibrillation

**DOI:** 10.34172/jcvtr.33269

**Published:** 2024-12-23

**Authors:** Hadi Charati, Ahmad Hamta

**Affiliations:** ^1^Department of Biology, Faculty of Sciences, Arak University, Arak, Iran; ^2^Center for Intelligent Medicine Research, Greater Bay Area Institute of Precision Medicine (Guangzhou), Fudan University, Guangzhou, China

**Keywords:** Atrial fibrillation, Cis-Pqtl, Drug targets, Mendelian randomization, Myocardial infarction

## Abstract

**Introduction:**

Plasma proteins play essential roles in myocardial infarction (MI) and atrial fibrillation (AF); however, it remains unknown whether the two disorders share causal plasma proteins.

**Methods:**

The present study utilizes cis-protein quantitative trait loci (cis-pQTLs) for 4,719 plasma proteins to assess their causality on MI and AF.

**Results:**

Two-sample Mendelian randomization (MR) identifies 21 and 9 plasma proteins for MI and AF, respectively (FDR *P*<0.05), with plasminogen (PLG) being a commonly protective factor against both diseases. Multi-trait MR suggests that PLG is also protective against coronary atherosclerosis. PheWAS analysis identifies associations of six *cis*-pQTLs with both MI and AF, i.e., rs11751347 (PLG), rs11591147 (PCSK9), rs77347777 (ITIH4), rs936228 (ULK3), rs2261033 (AIF1V), and rs2711897 (BDH2). Furthermore, interactions exist among the causal plasma proteins, with PLG directly interacting with multiple others. Drug-gene databases suggest that PLG activators, such as Urokinase, Reteplase, Streptokinase, Alteplase, Anistreplase, Tenecteplase, Desmoteplase, and Defibrotide sodium may serve as common therapeutic drugs for MI and AF.

**Conclusion:**

Our study provides a causal inference of human plasma proteins in MI and AF. Several of the identified proteins and single nucleotide polymorphisms (sNPs) exert pleiotropic effects on other cardiometabolic phenotypes, indicating their crucial roles in the pathology of cardiovascular disease (CVD). Our study provides new insights into the shared causality and drugs for MI and AF.

## Introduction

 Myocardial infarction (MI) is caused by lack of blood flow to a portion of the heart muscle, which results in symptoms such as chest pain, shortness of breath and arrhythmias.^1^ Notably, stress, high cholesterol levels, obesity, diabetes, smoking, and menopause are associated with an increased risk of MI.^2^ Medications such as aspirin and statins have been shown to reduce the risk of MI.^3,4^ Atrial fibrillation (AF) is defined as an irregular and often rapid heart rhythm, which can increase the risk of blood clots forming in the heart. AF increases the risk of both MI and stroke.^5,6^ In the patients with AF, the incidence of MI is approximately 50% higher than those without and AF coexists in 6-21% of patients with acute MI.^7-9^ Additionally, patients with a history of hypertension are at an increased risk of developing AF.^10^ Observational studies have shown associations between MI and AF, with several proposed mechanisms. For instance, MI can lead to left atrial dilation, left ventricular remodeling, electrical remodeling, neurohumoral modulation, and loss of cardiomyocytes due to apoptosis and fibrosis. conversely, AF can result in thrombus formation and coronary artery embolism, as well as a mismatch between oxygen supply and demand caused by the arrhythmias.^5^, ^7^, ^11^, ^12^ Additionally, inflammation has been proposed as a common risk factor, with inflammatory mediators such as TNF, IL-2, and TGF-β1 playing critical roles.^7^ While comorbidity between MI and AF has been established, their molecular connections—particularly shared causal molecules—require further elucidation.

 Circulating proteins in the plasma, which include simple proteins, glycoproteins, lipoproteins, and other conjugated proteins, play essential roles in human physiology and pathology. They contribute to various functions, including the maintenance of colloid osmotic pressure, blood clotting, immune response, hormone transport and interorgan communication. Measurement these proteins provides insights into metabolic and inflammatory status, contributing to an overall assessment of human health.^13^ Nowadays, many drugs are designed to specifically target plasma proteins.^14^

 With the rapid advancement of genome-wide association studies (GWAS), the Mendelian randomization (MR) analytical strategy has become widely utilized to evaluate causal relationships between risk factors (exposure) and diseases (outcome) through genetic variants.^15^ Utilizing protein quantitative trait loci (pQTLs) as instruments, MR analysis has identified plasma proteins that are causal for complex diseases, including cardiovascular disease (CVD),^16-18^ such as stroke,^19^ heart failure,^20,21^ coronary atherosclerosis,^22,23^ and hypothyroidism.^24^ Zheng et al (2020) estimated the effects of 1,002 proteins on MI based on 738 *cis*-pQTLs and identified putatively causal effects of 90 *cis*-pQTLs on MI.^23^ Ning et al (2023) identified 30 proteins as potential drug targets for AF using 1,949 proteins, each represented by a single nucleotide polymorphisms (SNP) as instruments.^25^

 In the present study, we applied MR analytics to a large genetic study on pQTLs in the human plasma proteome from deCODE, encompassing 4,719 proteins, along with two most recent GWAS on MI and AF.^26-28^ We identified causal plasma proteins for MI and AF and analyzed their functions and pleiotropic effects on other CVD. We localized their shared causal plasma proteins and subsequently identified common drug candidates for the treatment of both diseases.

## Materials and Methods

###  Data resources

 All input datasets consisted of publicly available GWAS summary statistics conducted within the European ancestry group. For the MR analysis, exposure data was obtained from deCODE (https://www.decode.com/summarydata/), which included pQTLs for 4,719 plasma proteins in a cohort of 35,559 Icelanders. These proteins were measured using 4,907 aptamers via the Somascan v4 array. Outcome information for MI and AF was sourced from the Open GWAS project (https://gwas.mrcieu.ac.uk/). Specifically, the GWAS summary results for MI included association tests for 10,290,368 SNPs conducted between 14,825 cases and 2,680 controls (https://www.ebi.ac.uk/gwas/studies/GCST011365). The GWAS summary statistics for AF included association tests for 12,095,506 variants, involving 55,114 cases and 482,295 controls (https://gwas.mrcieu.ac.uk/datasets/ebi-a-GCST006061/).

###  Mendelian randomization analysis

 The “TwoSampleMR” R package (version 0.5.6) was utilized to conduct the MR analysis, employing only summary statistics from the GWAS.^29^ The exposure and outcome data were loaded and harmonized using the harmonise_data function. To ensure the independence of the instruments for the exposure, we employed the clump_data function to clump SNPs, referencing the European samples from the 1000 Genomes Project.^29^ Five MR methods, namely inverse variance weighted (IVW),^30^ MR Egger, weighted median,^31^ Simple mode, Weighted mode,^32,33^ and Wald ratio were used to analyze the causal association of *cis*-pQTLs with MI and AF. FDR-corrected *P* values were calculated, with significance determined at an FDR threshold of < 0.05.^34^

###  Enrichment analysis

 Functional and pathway enrichment analyses based on Gene Ontology (GO) and the Kyoto Encyclopedia of Genes and Genomics (KEGG) was conducted using the DAVID bioinformatics tool (https://david.ncifcrf.gov/). In this analysis, the proteins identified in the MR analysis were utilized as input data. *P* value < 0.05 was considered significant for GO and KEGG pathway enrichment.

###  Multitrait mendelian randomization analysis

 Multi-trait MR was conducted using cis-pQTLs as the exposure and GWAS summary statistics for thirteen cardiovascular diseases as the outcomes (Supplementary Table S1).^29^ An association was deemed significant if it passing the threshold of FDR < 0.05. The results were visualized as a dotplot using the ggplot2 package in R.

###  phenome-wide association studies (PheWAS)

 PheWAS analysis was conducted using Open Targets Genetics (https://genetics.opentargets.org) to investigate associations between SNPs and CVDs in a large numbers of individuals. For each of the thirty SNPs identified as causal for MI and AF in our MR analysis, we conducted independent PheWAS. statistical significance was established at a threshold of *P* < 0.005. A manhattan plot was utilized to visualize the results.

 Open Targets Genetics is an open-access web resource that explores associations between GWAS-associated loci, variants, traits, and causal genes.

###  Drug target analysis 

 To derive potential drugs for each plasma protein candidate identified in our MR findings, we referenced an updated list from the Drug Gene Interaction Database (DGIdb).^35^ We conducted a manual search for drug targets through an inquiry into https://dgidb.org/ (DGIdb) website for each candidate target.

###  Protein-protein interaction (PPI) network analysis

 To investigate the relationships between the causal plasma proteins for MI and AF, we conducted a PPI network analysis using the String software (http://string-db.org/).^36^ This analysis searched the PPI records in the String database and subsequently constructed a PPI network for all identified causal proteins.

## Results

###  Causal plasma proteins to MI and AF

 We performed two-sample MR using various methods to identify causal plasma proteins associated with MI and AF. This analysis utilized the pQTL dataset of the human plasma proteome from deCODE, along with data from two large GWAS on MI and AF.^26-28^ The deCODE pQTL dataset comprises 28,191 associations between DNA genotypes and the levels of 4,719 plasma proteins. This includes 7,572 SNPs located near the protein-coding genes, classified as cis-pQTL. Since *cis*-pQTLs typically exert greater effects on protein abundance than *trans*-pQTLs,^22^ we concentrated on *cis*-pQTLs for the subsequent analysis. The GWAS studies on MI and AF, each examined over 10 million SNPs in disease cohorts comprising 14,000 MI cases and 55,000 AF cases, making them the largest genetic studies of their kind. All these studies were conducted on populations of European ancestry, resulting in large and homogenous datasets for the subsequent discovery.

**Figure 1:**
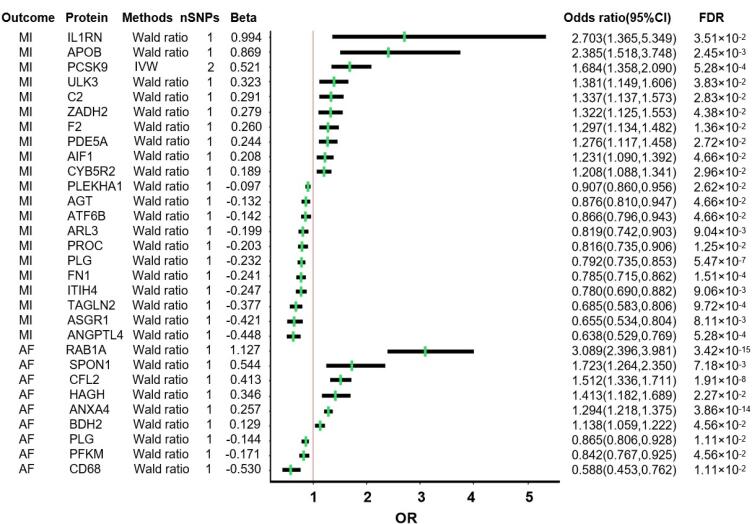
Mendelian randomization analysis of plasma proteins (cis-pQTLs) for MI and AF. The forest plot displays significant causal effects of plasma proteins on MI and AF. The green lines denotes the averaged odds ratio and the error bars indicate the 95% confidence interval

 Overall, our MR analysis identified 21 proteins associated with MI (Inverse variance weighted or Wald Ratio MR, FDR < 0.05), including 10 proteins whose elevated expression would cause MI (interleukin-1 receptor antagonist (IL-1RN), apolipoprotein B (APOB), proprotein convertase subtilisin/kexin type 9 (PCSK9), unc-51 like kinase 3 (ULK3), complement C2 (C2), zinc binding alcohol dehydrogenase domain containing 2 (ZADH2), coagulation factor II (F2), phosphodiesterase 5A (PDE5A), allograft inflammatory factor 1 (AIF1), and cytochrome B5 reductase 2 (CYB5R2)) and 11 proteins whose expression would exert protective effects (pleckstrin homology domain containing A1 (PLEKHA1), angiotensinogen (AGT), activating transcription factor 6 beta (ATF6B), ADP-ribosylation factor-like protein 3 (ARL3), protein C (PROC), plasminogen (PLG), fibronectin 1 (FN1), inter-alpha-trypsin inhibitor heavy chain 4 (ITIH4), transgelin 2 (TAGLN2), asialoglycoprotein receptor 1 (ASGR1), and angiopoietin-like 4 (ANGPTL4)) (Figure 1, Supplementary Table S2). Notably, PCSK9 on Chromosome 1, a recognized biomarker for CVD, was identified as causal through two SNPs: rs11591147 and rs472495 (OR:1.68, FDR = 5.28 × 10^-4^). The plasma protein with the most significant causal effect on MI was IL-1RN (OR:2.70, 95%CI:1.36–5.35, FDR = 3.51 × 10^-2^).

 Concurrently, we identified 9 causal plasma proteins associated with AF (Wald ratio MR, FDR < 0.05), of which 6 proteins were found to increase the risk of AF (member RAS oncogene family (RAB1A), spondin 1 (SPON1), cofilin-2 (CFL2), hydroxyacylglutathione hydrolase (HAGH), annexin A4 (ANXA4), and 3-hydroxybutyrate dehydrogenase 2 (BDH2)) and three proteins decrease the AF risks (cluster of differentiation 68 (CD68), phosphofructokinase, muscle (PFKM), and PLG) (Figure 1, Supplementary Table S3). The proteins with the most significant positive and negative effects on AF were RAB1A (OR:3.09, 95%CI:2.40–3.98, FDR = 3.42 × 10^-15^) and CD68 (OR:0.59, 95%CI:0.45–0.76, FDR = 1.11 × 10^-2^), respectively.

 Taken together, our MR analyses identified 29 plasma proteins as causal, with PLG being implicated in both MI (OR:0.79, 95% CI:0.74–0.85, FDR = 5.47 × 10^-7^) and AF (OR:0.86, 95%CI:0.81–0.93, FDR = 1.11 × 10^-2^). These results suggest that a decrease in the plasma level of PLG may increased the risks for both MI and AF.

###  Functional enrichments to infer biological mechanisms

**Figure 2:**
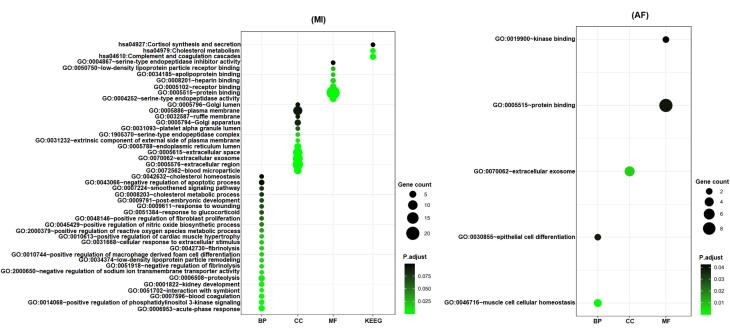
GO enrichment and KEGG pathway analysis for causal plasma proteins for MI and AF

 These potentially causal plasma proteins may reflect essential biological mechanisms underlying the diseases. To gain deeper insights, we conducted a functional enrichment analysis. For MI, the most significantly enriched terms included the acute-phase response, which was represented by four proteins (IL-1RN, FN1, F2, and ITIH4) (*P* = 9.02 × 10^-6^), blood microparticle which was represented by six proteins (PLG, FN1, F2, ITIH4, AGT, and ANGPTL4) (*P* = 1.74 × 10^-7^), extracellular region which was represented by twelve proteins (PLG, ASGR1, FN1, F2, APOB, TAGLN2, PROC, ITIH4, AGT, PCSK9, ANGPTL4, and C2) (*P* = 4.91 × 10^-7^), serine-type endopeptidase activity which was represented by four proteins (PLG, F2, PROC, PCSK9, and C2) (*P*= 3.62 × 10^-5^), and complement and coagulation cascades which were represented by four proteins (PLG, F2, PROC, and C2) (*P* = 2.95 × 10^-4^) (Figure 2, Supplementary Table S4). For AF, the most significant enrichments were found in muscle cell cellular homeostasis (*P* = 4.85 × 10^-5^) and extracellular exosome (*P* = 0.0065) (Figure 2, Supplementary Table S4).

###  Protein-protein interaction among the causal plasma proteins

**Figure 3:**
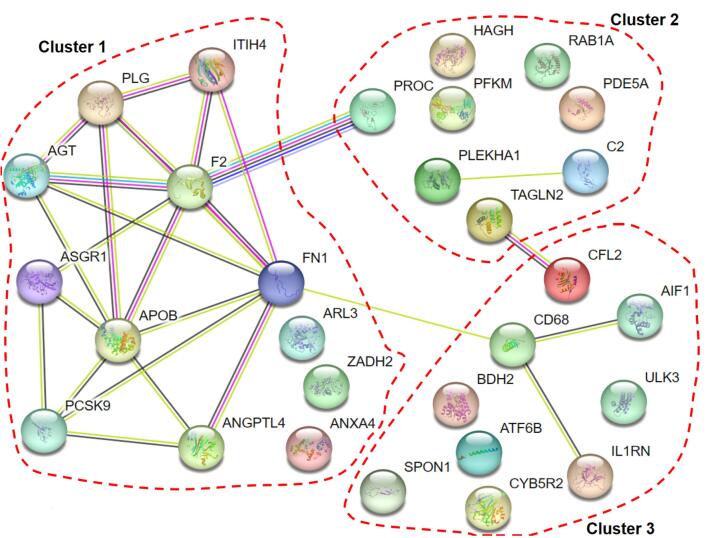
STRING-based PPI network of the 29 causal plasma proteins collectively identified for MI and AF. Colored nodes represent query proteins and the first shells of interactions. The drawn edges represent the existence of different types of evidence used in predicting the associations. The edges are coded as follows: Light blue, known interaction curated from databases; pink, known interaction determined through experiments; green, predicted interaction in the gene neighborhood; red, gene fusions; dark blue, gene co-occurrence; lime green, text mining; black, co-expression; indigo, protein homology. Clusters are indicated with red dashed lines

 We constructed a PPI network for 29 causal plasma proteins associated with MI or AF using STRING (http://string-db.org/) at a medium confidence level (0.4) (Figure 3). CD68, which is protective against AF, directly interacted with FN1 (protective against MI), AIF1 (causal to MI), and IL-1RN (causal to MI). CFL2, causal to AF, interacted directly with TAGLN2 (causal to MI). PLG, protective against both MI and AF, primarily interacted with MI related proteins. k-means clustering was employed to identify sub-networks with similar topological and functional properties among the proteins, resulting in three distinct clusters. Cluster 1 included PLG and eleven other causal proteins, with KEGG pathway analysis revealing enrichment for cholesterol metabolism (*P* = 0.001) (Supplementary Table S5). No pathway enrichment was identified for the other two clusters.

###  Multi-trait MR identified causal proteins for multiple cardiometabolic phenotypes

**Figure 4:**
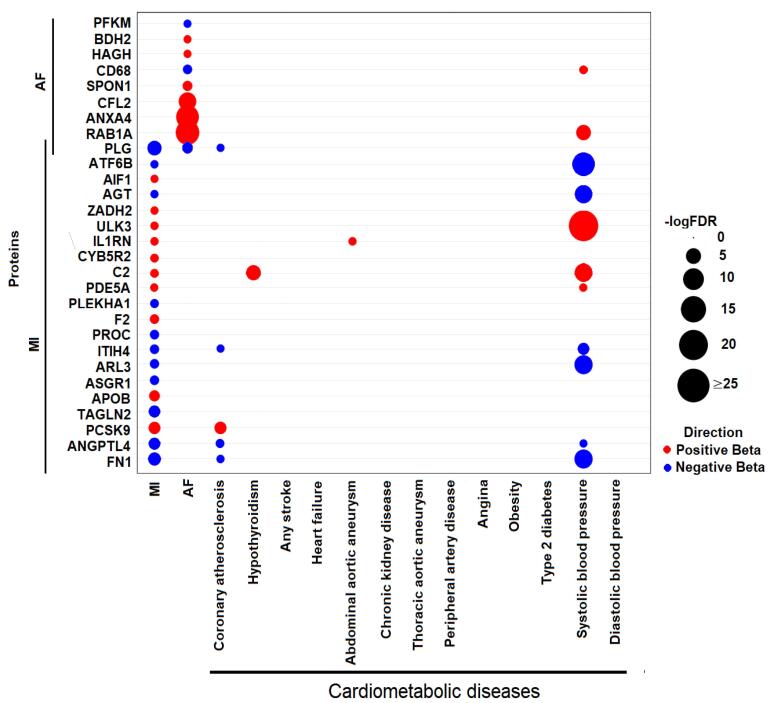
Multi-trait MR analysis to evaluate pleiotropic effects of the 29 plasma proteins. The plasma proteins were used as exposures and fifteen cardiovascular phenotypes, including MI and AF in the initial analysis, were set as outcomes. Red and blue indicate positive and negative directional effects respectively

 Cardiovascular and metabolic diseases share common risk factors. To determine whether the 29 causal plasma proteins exhibit pleiotropy for other cardiometabolic phenotypes, we conducted a multi-trait MR analysis. We gathered GWAS summary statistics for thirteen additional cardiometabolic phenotypes, including coronary atherosclerosis, hypothyroidism, stroke, heart failure, angina, abdominal aortic aneurysm, thoracic aortic aneurysm, peripheral artery disease, chronic kidney disease, type 2 diabetes, obesity, systolic blood pressure, and diastolic blood pressure (Figure 4, Supplementary Table S1). Interestingly, PLG, ITIH4, FN1, and ANGPTL4, all protective against MI, were also inferred to be protective against coronary atherosclerosis. Conversely, PCSK9, which is causal to MI, was similarly inferred to be causal for coronary atherosclerosis. The consistent directions of effect underscore the significant roles of these plasma proteins. Additional causal relationships identified included C2 for hypothyroidism and IL-1RN for abdominal aortic aneurysm (Figure 4). Notably, striking causal relationships between systolic blood pressure and eleven causal plasma proteins associated with AF and MI are also evident in Figure 4.

###  PheWAS identified pleiotropic effects of the SNPs of MI and AF

**Figure 5:**
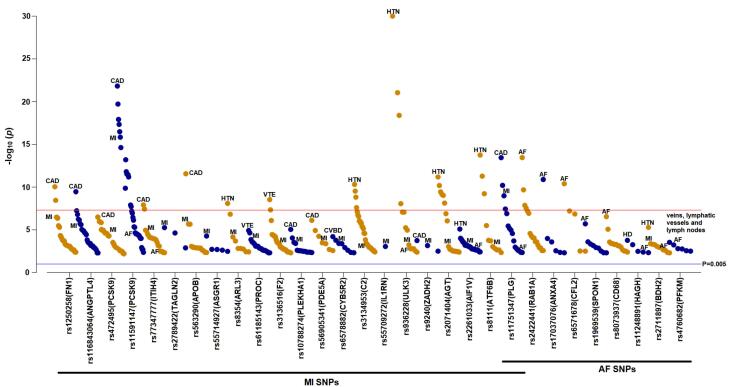
PheWAS analysis for MI or AF related SNPs that are also *cis*-pQTLs. The Manhattan plot shows the association between 30 SNPs and cardiovascular diseases in the UK Biobank, FinnGen and GWAS Catalog summary statistics repository. On the X axis are SNPs associated with MI or AF that are also *cis*-pQTLs for plasma proteins. The Y axis denotes the PheWAS association P-values for each ascertained phenotype. The blue horizontal line represents P = 0.005 and the red horizontal line represents P = 1 × 10^-5^. MI: Myocardial infarction; AF: Atrial fibrillation; CAD: Coronary artery disease; HTN: Hypertension; VTE: Venous thromboembolism; CVBD: Cerebrovascular disease; HD: Heart disease

 We also examined the pleiotropic effect of the SNPs associated with MI or AF on other cardiovascular diseases (Figure 5, Supplementary Table S6). The most significant association was observed between rs936228 (ULK3) and hypertension (*P* = 1 × 10^-30^). Most MI-associated SNPs were also linked to coronary artery disease and hypertension. Notably, six SNPs, namely rs11591147 (PCSK9), rs77347777 (ITIH4), rs936228 (ULK3), rs2261033 (AIF1V), rs11751347 (PLG), and rs2711897 (BDH2) were found to be associated with both MI and AF.

###  Candidate drug targets for MI and AF

 With the elucidation of the causality of plasma proteins for MI and AF, we queried the Drug Gene Interaction Database (DGIdb)^35^ for potential drug candidates that could correct abnormal levels of the causal proteins (Table 1, Supplementary Table S7). Among the 27 drugs targeting PLG, eight activators (Urokinase, Reteplase, Streptokinase, Alteplase, Anistreplase, Tenecteplase, Desmoteplase, and Defibrotide sodium) were particularly noteworthy, as low PLG levels were inferred to be causal for both MI and AF. Likewise, four PCSK9 inhibitors (alirocumab, bococizumab, evolucumab, and RG-7652), along with a ULK3 inhibitor (Hesperadin), a PROC activator (Menadione), and ten PDE5A inhibitors (Udenafil, Dipyridamole, Tadalafil, Vardenafil, Sildenafil, Pentoxifylline, Vardenafil hydrochloride, Avanafil, Sildenafil citrate, and Theophylline) were identified as potential drugs to correct the elevated expression of these proteins. Other listed drugs require further assessments, as their interaction types or drug effects were not provided.

**Table 1:** Candidate drug targets for MI and AF from Drug-Gene Interaction Database (DGIdb)

**Disease**	**Proteins**	**Interaction types**	**Drug**
AF and MI	PLG	Activator	UROKINASE, RETEPLASE, STREPTOKINASE, ALTEPLASE, ANISTREPLASE, TENECTEPLASE, DESMOTEPLASE, DEFIBROTIDE SODIUM
		Inhibitor	TRANEXAMIC ACID,APROTININ, AMINOCAPROIC ACID
		Not provided	RANOLAZINE, GARLIC, GENISTEIN, NYSTATIN, AMEDIPLASE, INOSITOL, MELAGATRAN, CHEMBL35482, NORETHINDRONE, FIBRINOLYSIN, OXYMETHOLONE, BORTEZOMIB, HUMAN GROWTH HORMONE, LEPIRUDIN, RADIOSUMIN B, COUMARIN, PREDNISOLONE, DANAZOL,
MI	FN1	Cleavage	OCRIPLASMIN
		Not provided	L19IL2, L19TNFA, L19SIP 131I
	PCSK9	Inhibitor	ALIROCUMAB, BOCOCIZUMAB, EVOLOCUMAB, RG-7652
		Not provided	FROVOCIMAB, LOMITAPIDE
	APOB	Not provided	EPIGALOCATECHIN GALLATE, QUERCETIN, ATORVASTATIN, DEXAMETHASONE, IRBESARTAN, VITAMINE, ALCOHOL, MIPOMERSEN, NEVIRAPINE, LOVASTATIN, HEPARIN, LOMITAPIDE, HYDROCORTISONE, CHOLESTYRAMINE, WARFARIN, PRAVASTATIN, TRIFLUOPERAZINE, FENOFIBRATE
	PROC	Activator	MENADIONE
		Not provided	WARFARIN, PROGESTERONE, PHENPROCOUMON, AVATROMBOPAG, ANCROD, LUSUTROMBOPAG, ESTRADIOL
	F2	Activator	MENADIONE
		Inhibitor	LEPIRUDIN,ARGATROBAN, XIMELAGATRAN, DESIRUDIN, MELAGATRAN, DABIGATRAN, DABIGATRAN ETEXILATE MESYLATE, BIVALIRUDIN
		Not provided	AVATROMBOPAG, APRAMIDE A, LUSUTROMBOPAG, PEGMUSIRUDIN, SILIBININ, QUERCETIN, ENOXAPARIN, HEPARIN, CIANIDANOL, AZD-8165, BORTEZOMIB, ANISINDIONE, ODIPARCIL, CYANIN, TAMOXIFEN, CYANIDIN, EPICATECHIN, ATECEGATRAN METOXIL, CHEMBL586628, CHEMBL1083499
	PDE5A	Inhibitor	UDENAFIL, DIPYRIDAMOLE, TADALAFIL, VARDENAFIL, SILDENAFIL, PENTOXIFYLLINE, VARDENAFIL HYDROCHLORIDE, AVANAFIL, SILDENAFIL CITRATE, THEOPHYLLINE
		Not provided	RHUCIN, EXISULIND, ICARIIN, ROLIPRAM,CP 461, CHEMBL460293, LORNEIC ACID A, PAPAVERINE, ENALAPRIL
	CYB5R2	Not provided	RASBURICASE, PRIMAQUINE, METOCLOPRAMIDE
	IL1RN	Not provided	METHOTREXATE, HALOPERIDOL, DIACEREIN
	ULK3	Inhibitor	HESPERADIN
		Not provided	IMATINIB
	AGT	Not provided	ATENOLOL, ADRIAMYCIN, ENALAPRIL, AMLODIPINE, HYDROCORTISONE, CHLORTHALIDONE, ASPIRIN, LISINOPRIL, CYT006-ANGQB, QUINAPRIL, IRBESARTAN, MESTRANOL, BENAZEPRIL, IMIDAPRIL

MI, myocardial infarction; AF, atrial fibrillation; PLG, plasminogen; FN1, fibronectin 1; PCSK9, proprotein convertase subtilisin/kexin type 9; APOB, apolipoprotein B; PROC, protein C; F2, coagulation factor II; PDE5A, phosphodiesterase 5A; CYB5R2, cytochrome B5 reductase 2; IL1RN, interleukin-1 receptor antagonist; ULK3, unc-51 like kinase 3; AGT, angiotensinogen

## Discussion

**Figure 6:**
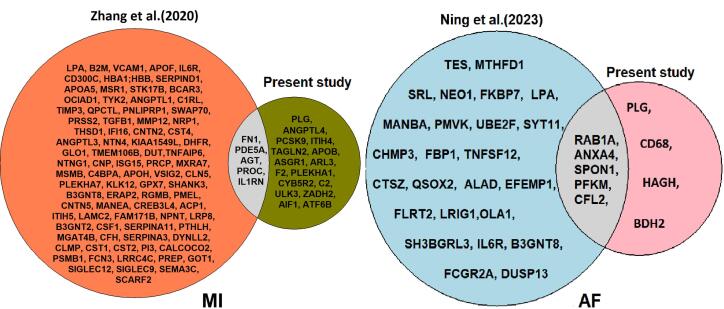
Comparison of MI and AF causal blood proteins found in the present study with previous studies

 In the present study, we utilized *cis*-pQTLs for human plasma proteins and GWAS summary statistics for MI and AF to performed two-sample MR analyses, inferring the causality of plasma proteins in relation to these diseases. We identified a total of 21 plasma proteins associated with MI, 9 associated with AF, and one protein, PLG, that is commonly protective against both diseases. While five of these plasma proteins have been reported in previous MR studies (Figure 6),^23,25^ the remaining 16 proteins for MI and 4 proteins for AF are novel findings, highlighting the discovery potential of a larger *cis*-pQTL dataset.

 We performed a PPI analysis for the causal proteins identified in this study (Figure 3). Previous studies have demonstrated that genes associated with similar disorders or phenotypes tend to encode proteins that interact with one another through PPIs.^37,38^ Thus, this analysis aims to enhance our understanding of the relationship between MI and AF and their complex biology.^38^ While many plasma proteins associated with MI and AF interacted with each other, nearly half did not have direct connections, and some were identified as orphan proteins within our network. Further investigations are required to uncover potential links through mediating proteins.

 PLG is the common plasma protein identified in our analysis for both MI and AF. It plays a crucial role in the fibrinolytic process and regulates the migration of immune cells to site of inflammation, thereby playing a prominent role in cardiovascular pathology. to the best of our knowledge, its protective effect against the risk of MI has been attributed to its association with stem cell-mediated cardiac repair; however, its role in AF has not yet been reported.^39^

 It is worth mention that nine plasma proteins associated with MI also displayed causality for systolic blood pressure in our multi-trait MR analysis. Previous studies have identified hypertension as a risk factor for MI, coronary heart disease, and stroke, with evidence showing that controlling hypertension can reduce the incidence of fatal MI and stroke.^40^ Additionally, IL-1RN, a member of the interleukin 1 cytokine family, was inferred to be causal for both MI and abdominal aortic aneurysm in our study. IL-1RN is linked to classic pro-inflammatory cytokine and plays a pivotal role in the innate immune response.^41^ A recent study indicated that IL-1 blockade is associated with a lower risk of CVDs in patients with prior acute MI.^42^ Another study suggested that inhibiting interleukin-1 could be a potential strategy to protect against abdominal aortic aneurysm in hypertensive patients.^43^ Our results align with these findings, highlighting the need for further investigation into the role of IL-1RN. Additionally, our analysis identified PCSK9 as causal for MI, with PCSK9 inhibitors such as Evolocumab, Alirocumab, RG.7652, and Bococizumab emerging as candidate drugs for treating MI.^44^ This is consistent with previous studies showing that PCSK9 inhibitors reduced the risk of coronary atherosclerosis.^45,46^

## Conclusion

 Our study provides causal inference regarding human plasma proteins in relation to MI and AF. Several of the identified proteins and SNPs exhibit pleiotropic effects on other cardiometabolic phenotypes, indicating their essential roles in CVD pathology. We suggest potential drugs for these plasma proteins, particularly PLG activators, such as Urokinase, Reteplase, Streptokinase, Alteplase, Anistreplase, Tenecteplase, Desmoteplase, and Defibrotide sodium, as potential therapeutic drugs for treating comorbid MI and AF. Our study provides a new hatch toward understanding the shared causality and drugs for MI and AF.

## Acknowledgments

 We would like to express our gratitude to the members of the Institute of Precision Medicine at Fudan University (Guangzhou) for their support.

## Competing Interests

 There are no conflicts of interest to declare.

## Ethical Approval

 The authors declare no ethical conflicts. All datasets used in this study were publicly available online from deCODE (https://www.decode.com/summarydata/) and the Open GWAS project (https://gwas.mrcieu.ac.uk/).

## Supplementary File

Supplementary file contains Tables S1-S7.
